# Factors Associated With Parental Acceptance of Minimally Invasive Tissue Sampling to Identify the Causes of Stillbirth and Neonatal Death

**DOI:** 10.1093/cid/ciab829

**Published:** 2021-12-15

**Authors:** Shiyam Sunder Tikmani, Sarah Saleem, Janet L Moore, Sayyeda Reza, Guruprasad Gowder, Sangappa Dhaded, S Yogesh Kumar, Shivaprasad S Goudar, Vardendra Kulkarni, Sunil Kumar, Anna Aceituno, Lindsay Parlberg, Elizabeth M McClure, Robert L Goldenberg

**Affiliations:** 1 Department of Community Health Sciences, Aga Khan University, Karachi, Pakistan; 2 RTI International, Durham, North Carolina, USA; 3 JJM Medical College, Davengere, India; 4 KLE Academy of Higher Education and Research’s Jawaharlal Nehru Medical College, Belagavi, India; 5 Department of Obstetrics and Gynecology, Columbia University School of Medicine, New York, New York, USA

**Keywords:** Acceptance, Minimally invasive tissue sampling, neonatal death, Stillbirth

## Abstract

**Background:**

Minimally invasive tissue sampling (MITS) is a noninvasive technique used to determine the cause of deaths. Very little is known about the factors that affect MITS acceptance or refusal. We present findings from a prospective study conducted in Southeast Asia on the reasons for accepting or refusing MITS.

**Methods:**

This substudy was conducted in India and Pakistan to determine the acceptability of MITS in women who had a stillbirth or preterm live birth who later died. A formal questionnaire was used to gather observations during the consent for MITS, such as reasons for acceptance or refusal of MITS, as well as which family members were involved in the decision process.

**Results:**

In Pakistan, the MITS acceptability forms were completed for 470 of 477 women (98.5%) with an eligible stillbirth for this substudy, and 334 of 337 (99.1%) with an eligible preterm neonatal death. In India, MITS acceptability forms were completed in 219 of 305 women (71.8%) with an eligible stillbirth and 260 of 264 (98.4%) with an eligible preterm neonatal death. In India, the most common reasons for MITS refusal for both stillbirths and preterm neonatal deaths were cultural concerns, while in Pakistan, the most common reason for MITS refusal was a potential delay in the funeral. The primary reason for accepting MITS was that the parents wanted to understand the cause of death. At both sites, fathers, mothers, and relatives, often in consultation, choose whether or not to accept MITS to determine the cause of death in stillbirths and preterm neonatal deaths.

**Conclusions:**

MITS was more commonly accepted in India than in Pakistan. Cultural concerns in India and funeral delays in Pakistan were common reasons for refusal. Parents from both sites were curious to know the cause of stillbirths and preterm neonatal deaths. The father, mother, and relatives were key decision makers for consenting to or declining MITS.

In many low-and middle-income countries, especially in south Asia and sub-Saharan Africa, despite governmental efforts, the stillbirth and neonatal mortality rates remain high [1]. To reduce the burden of stillbirths and neonatal deaths in low-and middle-income countries, it is imperative to determine the factors leading to these high mortality rates.

The standard methods of identifying the cause of death have certain challenges. For deaths occurring at home, there is often too little information to assign the cause of death [2]. Verbal autopsy is a commonly used tool to identify the causes of stillbirth and neonatal death. The limitations of this approach, based on an interview with a mother or family members on clinical events leading to stillbirth and neonatal deaths, include incomplete data, recall bias, and subjectivity of physicians who assign the cause of death based on verbal autopsy [2].

The reference standard for assessing the cause of death in institutional deaths is complete diagnostic autopsy (CDA) [3]. However, CDA is rarely performed in low-and middle-income countries, for reasons including limited human resources, particularly of trained personnel with the technical expertise required to carry out this procedure [4]. CDA is relatively expensive to perform, and in many areas the needed resources are not available. In addition, CDA may be refused for cultural and/or religious reasons [5, 6]. As a consequence, effective public health initiatives to reduce stillbirth and neonatal death are not optimal as the causes of death often remain unknown. As an alternative to CDA, minimally invasive tissue sampling (MITS), also called minimally invasive autopsy, may be a more suitable option to determine the causes of death in stillbirths and neonatal deaths [7]. The MITS procedure involves using biopsy needles to collect tissue samples (eg from the liver, lungs, and brain) and also may collect body fluids (eg, heart blood and cerebrospinal fluid). Because there are no incisions, there is no body disfigurement. Therefore, MITS may be an alternate acceptable procedure to inform the causes of fetal and neonatal deaths [4, 8].

The hypothetical acceptability of MITS to parents and relatives, as a way to learn cause of death, has been reported in some qualitative studies [6]. In a multicountry mixed-method study, including Pakistan, the overall hypothetical acceptability of MITS to determine the cause of death was 73%, slightly lower (54.3%) in Pakistan [6].

In a qualitative study to investigate healthcare providers’ perspectives on the acceptability of the MITS procedure, most healthcare providers believed that the MITS procedure might be appropriate for parents owing to its lack of disfigurement and that it would be preferred by parents who had already experienced unexplained infant death or stillbirth [9]. In another qualitative study, effective counseling, building trust with parents, fast procedure time, and approaching families within a few hours of death were believed to facilitate the acceptability of MITS [10, 11]. Couples who had experienced multiple stillbirths, neonatal deaths, and miscarriages were believed to be more likely to accept the MITS procedure [10].

We present the results of a prospective study on the reasons for acceptance or refusal of the MITS procedure from a study conducted in south Asia to understand the cause of death of stillbirths and preterm neonatal deaths, known as the Project to Understand and Research Preterm Pregnancy Outcomes and Stillbirths in South Asia (PURPOSe) [12].

## METHODS

### Study Setting and Design

PURPOSe, a prospective observational study, was carried out in Pakistan and India. In India, the study recruitment took place in 3 hospitals in Davengere, Karnataka, south India. In total, these hospitals have about 16 000 deliveries per year, with about 30% preterm births. The estimated rate of stillbirths was 25 per 1000 births. In Pakistan, recruitment took place in 2 major public sector tertiary care hospitals: Jinnah Post Graduate Medical Center (JPMC) and the National Institute of Child Health in Karachi, Pakistan. JPMC has about 15 000 deliveries per year with about 35% preterm births, while the estimated rate of stillbirths was 35 per 1000 births.

PURPOSe sought to determine the cause of death among stillbirths and preterm neonatal deaths, using MITS and other clinical data collected during the hospital or neonatal intensive care unit admission [12]. A senior research assistant enrolled women during labor or after delivery after obtaining initial consent to participate in the primary study on the causes of death.

For this substudy on the acceptability of MITS, women who delivered a stillborn infant or had a preterm live-born infant who later died were eligible. Observations during the consent for the MITS procedure, such as reasons for acceptance or refusal of MITS, and which family members participated in the decision process, were collected using a structured questionnaire.

Among all women with a stillbirth or preterm neonatal death, additional consent to undertake the MITS procedure was obtained from parents or caregivers in their native language. The consent to perform the MITS procedure was obtained from parents or caregivers but first required counseling by trained counselors. At both sites, the counselors, in addition to obtaining consent, also responded to questions raised from parents or guardians relating to the MITS procedure and cultural and religious beliefs. In a few cases, attending physicians also responded to questions from the parents [13].

### Questionnaire

The questionnaire was designed to determine whether MITS was acceptable to families that experience a stillbirth or preterm neonatal death and to determine the reasons for willingness or unwillingness to provide consent. The questionnaire was developed through a 2-step consultative process: (1) a literature review to identify factors that affect a general consent process and (2) the findings of a qualitative exploratory study to determine the hypothetical acceptability of the MITS procedure among parents, religious leaders, and healthcare providers [10, 11]. After the questionnaire was developed, the domain experts provided external review. Some questions required binary responses, and others required multiple responses.

### Statistical Analysis

All data were entered into a data management system at each of the study sites, where data quality checks were applied. Data were then transmitted to a central data management center, where additional edits were performed. Descriptive statistics summarized the variables of interest by site. Among deliveries with a stillbirth or neonatal death, univariate analyses were performed to determine the unadjusted relative risk (RR) and 95% confidence intervals (CIs) between the association of maternal and fetal/neonatal characteristics and the acceptance of MITS. SAS software (version 9.4; SAS Institute) was used for analysis. Because not all questions were answered by all respondents, at both sites, the denominators differ for some variables.

### Ethical Approval

The study was approved by the ethical review committees of Aga Khan University, JPMC, the National Institute of Child Health and the National Bioethics Committee (Karachi, Pakistan), KLE Academy of Higher Education and Research (Belagavi, India), and J. J. M. Medical College (Davangere, India) and at Research Triangle Institute (RTI International) (Durham, North Carolina). All women provided informed consent before participating in the study. PURPOSe was registered in ClinicalTrials.gov (NCT03438110).

## RESULTS

**Figure 1.**
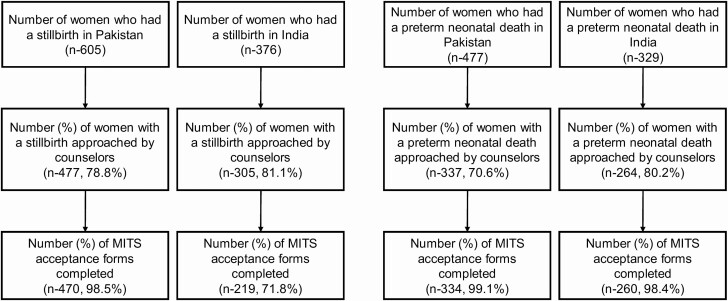
Flow diagram. Abbreviation: MITS, minimally invasive tissue sampling.

In Pakistan, during the study period, 605 women experience a stillbirth, and 477 a preterm neonatal death. Among those women, the counselors approached 477 (78.8%) with a stillbirth and 337 (70.6%) with a preterm neonatal death. The MITS acceptability forms were completed for 470 of 477 women (98.5%) approached with a stillbirth and 334 of 337 (99.1%) approached with a preterm neonatal death (Figure 1).

In India, during the study period, 376 women had a stillbirth, and 329 women had a preterm neonatal death. Of these, the counselors approached 305 women (81.1%) with a stillbirth and 264 (80.2%) with a preterm neonatal death. The MITS acceptability forms were completed for 219 of 305 women (71.8%) with a stillbirth who were approached and 260 of the 264 (98.4%) with a preterm neonatal death who were approached (Figure 1). At both sites, some parents refused to answer the MITS acceptability questions because they were in hurry to leave the hospital or were emotionally upset.

Among those who completed the questionnaire on MITS consent, we first evaluated the maternal demographics of those women with a stillbirth who consented or refused consent for MITS (Table 1). We did not observe statistically significant differences by maternal age at either site; however, at the Pakistani site, women with no formal schooling were more likely to consent to MITS than those with higher educational levels (RR, 1.49 [95% CI, 1.13–1.96]). We did not observe an increased likelihood to consent for MITS associated with prior abortion or stillbirth. Women with multiple pregnancies were more likely to consent to MITS at both sites, although the numbers were small. In Pakistan, having more antenatal care (ANC) visits was also associated with a higher likelihood of MITS refusal, compared with those with no ANC visits. At the Indian site, a greater likelihood of refusal was observed only for women having >4 visits. Finally, at the Pakistani site, we noted that women experiencing hemorrhage were more likely to consent to MITS than those with no hemorrhage (RR, 1.34 [95% CI, 1.06–1.69]).

**Table 1.** Maternal and Pregnancy Characteristics of Mothers With Stillbirth in Pakistan and India Approached for Minimally Invasive Tissue Sampling Evaluation

	India			Pakistan		
	Consented for MITS (SB), No. (%)			Consented for MITS (SB), No. (%)		
Characteristic	Yes (n = 179)	No (n = 37)	Unadjusted RR (95% CI)	Yes (n = 196)	No (n = 258)	Unadjusted RR (95% CI)
Maternal demographics						
Age group	n = 178	n = 37		n = 195	n = 258	
<20 y	12 (6.7)	2 (5.4)	1.04 (.83–1.30)	11 (5.6)	13 (5.0)	1.11 (.71–1.76)
20–30 y	149 (83.7)	32 (86.5)	1	130 (66.7)	186 (72.1)	1
>30 y	17 (9.6)	3 (8.1)	1.03 (.85–1.26)	54 (27.7)	59 (22.9)	1.16 (.92–1.47)
Educational level	n = 179	n = 37		n = 192	n = 254	
No formal schooling	28 (15.6)	3 (8.1)	1.09 (.94–1.25)	120 (62.5)	112 (44.1)	1.49 (1.13–1.96)
1–8 y	57 (31.8)	15 (40.5)	0.95 (.82–1.10)	31 (16.1)	65 (25.6)	0.93 (.64–1.36)
>8 y	94 (52.5)	19 (51.4)	1	41 (21.4)	77 (30.3)	1
Occupation	n = 179	n = 37		n = 195	n = 256	
Homemaker	162 (90.5)	34 (91.9)	1	192 (98.5)	247 (96.5)	1
Other	17 (9.5)	3 (8.1)	1.03 (.85–1.25)	3 (1.5)	9 (3.5)	0.57 (.21–1.53)
Pregnancy history						
Gravida	n = 179	n = 37		n = 195	n = 258	
Primigravida	78 (43.6)	20 (54.1)	0.93 (.82–1.05)	57 (29.2)	73 (28.3)	1.03 (.81–1.29)
Multigravida	101 (56.4)	17 (45.9)	1	138 (70.8)	185 (71.7)	1
Previous abortion	22 (21.8)	4 (23.5)	0.99 (.82–1.18)	46 (33.3)	67 (36.4)	0.92 (.71–1.21)
Previous stillbirth	10 (9.9)	2 (11.8)	0.97 (.75–1.26)	40 (29.0)	51 (27.6)	1.04 (.79–1.37)
Previous live birth	88 (87.1)	15 (88.2)	0.99 (.80–1.22)	123 (89.1)	162 (87.6)	1.09 (.72–1.66)
Current pregnancy characteristics						
Multiple pregnancy	3 (1.7)	0 (0.0)	1.21 (1.14–1.29)	8 (4.1)	4 (1.6)	1.57 (1.04–2.37)
ANC received	n = 179	n = 37		n = 194	n = 256	
None	1 (0.6)	0 (0.0)	1	41 (21.1)	24 (9.4)	1
1–3 visits	32 (17.9)	4 (10.8)	0.89 (.79–1.00)	69 (35.6)	91 (35.5)	0.68 (.53–.88)
≥4 visits	146 (81.6)	33 (89.2)	0.82 (.76–.87)	84 (43.3)	141 (55.1)	0.59 (.46–.76)
Clinical conditions						
Any hypertensive disorder	61 (34.1)	20 (54.1)	0.86 (.75–.99)	84 (43.1)	92 (35.8)	1.19 (.96–1.47)
Any antepartum hemorrhage	32 (17.9)	10 (27.0)	0.90 (.75–1.08)	44 (22.7)	37 (14.5)	1.34 (1.06–1.69)

Abbreviations: ANC, antenatal care; CI, confidence interval; MITS, minimally invasive tissue sampling; RR, relative risk.

In Table 2, we present the relationships between the mother’s demographic characteristics and consent for women with a preterm neonatal death. In Pakistan, lower educational levels were associated with a higher likelihood of consenting to MITS (RR, 1.73 [95% CI, 1.14–2.64]), while having more ANC visits was associated with a greater likelihood of refusing MITS. No other characteristic was significantly associated with acceptance or refusal of MITS.

**Table 2.** Maternal and Pregnancy Characteristics Among Mothers With Preterm Neonatal Deaths in Pakistan and India Approached for Minimally Invasive Tissue Sampling Evaluation

	India			Pakistan		
	Consented for MITS (ND), No. (%)			Consented for MITS (ND), No. (%)		
Characteristic	Yes (n = 162)	No (n = 68)	Unadjusted RR (95% CI)	Yes (n = 112)	No (n = 179)	Unadjusted RR (95% CI)
Maternal demographics						
Age group	n = 162	n = 68		n = 112	n = 178	
<20 y	17 (10.5)	5 (7.4)	1.11 (.87–1.42)	11 (9.8)	11 (6.2)	1.32 (.84–2.08)
20–30 y	133 (82.1)	58 (85.3)	1	82 (73.2)	135 (75.8)	1
>30 y	12 (7.4)	5 (7.4)	1.01 (.74–1.40)	19 (17.0)	32 (18.0)	0.99 (.66–1.46)
Educational level	n = 161	n = 67		n = 112	n = 179	
No formal schooling	8 (5.0)	5 (7.5)	0.83 (.53–1.28)	53 (47.3)	80 (44.7)	1.44 (.96–2.16)
1–8 y	51 (31.7)	27 (40.3)	0.88 (.73–1.06)	36 (32.1)	39 (21.8)	1.73 (1.14–2.64)
>8 y	102 (63.4)	35 (52.2)	1	23 (20.5)	60 (33.5)	1
Occupation	n = 162	n = 68		n = 112	n = 178	
Homemaker	154 (95.1)	65 (95.6)	1	107 (95.5)	170 (95.5)	1
Other	8 (4.9)	3 (4.4)	1.03 (.71–1.50)	5 (4.5)	8 (4.5)	1.00 (.49–2.01)
Pregnancy history						
Gravida	n = 162	n = 68		n = 112	n = 179	
Primigravida	71 (43.8)	32 (47.1)	0.96 (.81–1.14)	37 (33.0)	52 (29.1)	1.12 (.83–1.52)
Multigravida	91 (56.2)	36 (52.9)	1	75 (67.0)	127 (70.9)	1
Previous abortion	32 (35.2)	13 (36.1)	0.99 (.79–1.24)	30 (40.0)	42 (33.1)	1.20 (.84–1.73)
Previous stillbirth	11 (12.1)	3 (8.3)	1.11 (.82–1.50)	10 (13.3)	23 (18.1)	0.79 (.45–1.37)
Previous live birth	74 (81.3)	31 (86.1)	0.91 (.70–1.18)	65 (86.7)	113 (89.0)	0.88 (.53–1.46)
Current pregnancy characteristics						
Multiple pregnancy	19 (11.7)	5 (7.4)	1.14 (.91–1.43)	17 (15.2)	20 (11.2)	1.23 (.84–1.80)
ANC received	n = 162	n = 68		n = 112	n = 177	
None	0 (0.0)	0 (0.0)		13 (11.6)	11 (6.2)	1
1–3 visits	11 (6.8)	5 (7.4)	1	44 (39.3)	87 (49.2)	0.62 (.40–.96)
≥4 visits	151 (93.2)	63 (92.6)	1.03 (.73–1.44)	55 (49.1)	79 (44.6)	0.76 (.50–1.15)
Clinical conditions						
Any hypertensive disorder	62 (38.3)	18 (26.5)	1.16 (.99–1.37)	31 (27.7)	50 (27.9)	0.99 (.72–1.37)
Any antepartum hemorrhage	16 (9.9)	5 (7.4)	1.09 (.85–1.41)	19 (17.0)	25 (14.0)	1.14 (.79–1.66)

Abbreviations: ANC, antenatal care; CI, confidence interval; MITS, minimally invasive tissue sampling; RR, relative risk.

In Table 3, we present the characteristics of the stillbirths and preterm neonatal deaths to determine whether any were associated with the likelihood of consenting to MITS. In Pakistan, there was a higher likelihood (RR 1.37 [95% CI, 1.05–1.79]) of accepting MITS for multiple births and a higher likelihood of refusing MITS for preterm neonatal deaths with a lower birthweight (<1500 g) or a lower gestational age (<32 weeks). At the Indian site, we did not observe any differences in likelihood to consent for MITS associated with fetal or infant characteristics.

**Table 3.** Characteristics of Stillbirths and Preterm Neonatal Deaths

	India			Pakistan		
	Consented for MITS, No. (%)			Consented for MITS, No. (%)		
Characteristic	Yes	No	Unadjusted RR (95% CI)	Yes	No	Unadjusted RR (95% CI)
Characteristics of Stillbirths						
Stillbirths, no.	180	39		199	271	
Male sex	93 (51.7)	25 (64.1)	1.09 (.97–1.23)	107 (54.3)	123 (48.6)	0.88 (.71–1.09)
Multiple birth	4 (2.2)	2 (5.1)	0.81 (.46–1.43)	11 (5.5)	17 (6.3)	0.92 (.58–1.48)
Birthweight	n = 180	n = 39		n = 199	n = 257	
<1500 g	58 (32.2)	13 (33.3)	1.00 (.85–1.18)	55 (27.6)	150 (58.4)	0.46 (.34–.61)
1500–2499 g	74 (41.1)	15 (38.5)	1.02 (.88–1.19)	91 (45.7)	70 (27.2)	0.96 (.77–1.20)
≥2500 g	48 (26.7)	11 (28.2)	1	53 (26.6)	37 (14.4)	1
Gestational age	n = 180	n = 39		n = 173	n = 260	
≤32 wk	55 (30.6)	10 (25.6)	1.03 (.89–1.18)	59 (34.1)	138 (53.1)	0.56 (.42–.73)
32.0–36.6 wk	50 (27.8)	13 (33.3)	0.96 (.82–1.13)	59 (34.1)	75 (28.8)	0.82 (.63–1.06)
≥37 wk	75 (41.7)	16 (41.0)	1	55 (31.8)	47 (18.1)	1
Signs of maceration	86 (48.6)	15 (38.5)	1.08 (.95–1.22)	87 (44.2)	145 (56.4)	0.76 (.61–.94)
Characteristics of Preterm Neonatal Deaths						
Preterm neonatal deaths in facility, no.	186	74		136	198	
Male sex	107 (57.5)	42 (56.8)	0.99 (.85–1.16)	68 (50.4)	108 (55.1)	1.12 (.86–1.45)
Multiple birth	43 (23.1)	11 (14.9)	1.15 (.98–1.35)	41 (30.1)	39 (19.7)	1.23 (0.84, 1.80)
Birthweight	n = 185	n = 74		n = 136	n = 197	
<1500 g	140 (75.7)	47 (63.5)	1.31 (.69–2.50)	94 (69.1)	110 (55.8)	1.61 (.70–3.74)
1500–2499 g	41 (22.2)	24 (32.4)	1.10 (.57–2.15)	38 (27.9)	77 (39.1)	1.16 (.49–2.76)
≥2500 g	4 (2.2)	3 (4.1)	1	4 (2.9)	10 (5.1)	1
Gestational age	n = 186	n = 74		n = 136	n = 198	
≤32 wk	139 (74.7)	51 (68.9)	1.09 (.91–1.31)	112 (82.4)	161 (81.3)	1.04 (.74–1.47)
32.0–36.6 wk	47 (25.3)	23 (31.1)	1	24 (17.6)	37 (18.7)	1

Abbreviations: CI, confidence interval; MITS, minimally invasive tissue sampling; RR, relative risk.

Table 4 shows parents’ perceptions of MITS. In Pakistan, 271 women with a stillbirth and 198 with a preterm neonatal death refused the MITS procedure. The main reasons given for refusing the procedure in Pakistan were because it would delay the funeral (20.8% for stillbirths and 32.4% for preterm neonatal deaths), because the parents did not consider it necessary or useful (27.4% for stillbirths and 15.6% for preterm neonatal deaths), or because it was believed to be prohibited by the mother’s religion (12.3% for stillbirths and 7.3% for preterm neonatal deaths). In addition, a large proportion of parents with preterm neonatal deaths (21.2%) expressed mistrust in the hospital related to the MITS procedure. On the other hand, 115 parents with stillbirths (57.8%) consented to MITS because they wanted to know the cause of stillbirth. Similarly, 56.6% of parents with a preterm neonatal death wanted to know the cause of death; 33.2% of parents with a stillbirth and 27.9% with a preterm neonatal death did not specify any reason for consenting to MITS (Table 4).

**Table 4.** Parental Perceptions Related to Acceptance or Refusal of Minimally Invasive Tissue Sampling in Pakistan and India

	India				Pakistan			
	Consented for MITS (SB), No. (%)		Consented for MITS (ND), No. (%)		Consented for MITS (SB), No. (%)		Consented for MITS (ND), No. (%)	
Perceptions and Decision Process	Yes (n = 180)	No (n = 39)	Yes (n = 186)	No (n = 74)	Yes (n = 199)	No (n = 271)	Yes (n = 136)	No (n = 198)
Consent obtained for MITS	180 (100.0)	8 (20.5)	186 (100.0)	23 (31.1)	199 (100.0)	165 (60.9)	136 (100.0)	19 (9.6)
Consent not obtained	0 (0.0)	31 (79.5)	0 (0.0)	51 (68.9)	0 (0.0)	106 (39.1)	0 (0.0)	179 (90.4)
Reasons for not consenting								
Concern about funeral delay	…	11 (35.5)	…	8 (15.7)	…	22 (20.8)	…	58 (32.4)
Procedure considered unnecessary or not useful	…	6 (19.4)	…	11 (21.6)	…	29 (27.4)	…	28 (15.6)
Perceived prohibition by mother’s religion	…	2 (6.5)	…	6 (11.8)	…	13 (12.3)	…	13 (7.3)
Cultural considerations	…	18 (58.1)	…	32 (62.7)	…	6 (5.7)	…	10 (5.6)
Logistical issues	…	0 (0.0)	…	1 (2.0)	…	9 (8.5)	…	13 (7.3)
Fears related to removal of organs	…	0 (0.0)	…	0 (0.0)	…	2 (1.9)	…	5 (2.8)
Concerns about body disfigurement	…	2 (6.5)	…	5 (9.8)	…	2 (1.9)	…	12 (6.7)
Concern about unexpected medical findings	…	0 (0.0)	…	0 (0.0)	…	0 (0.0)	…	4 (2.2)
Lack of trust in hospital	…	0 (0.0)	…	2 (3.9)	…	3 (2.8)	…	38 (21.2)
Other reasons	…	3 (9.7)	…	4 (7.8)	…	37 (34.9)	…	41 (22.9)
Reasons for consenting to MITS								
Mother or father wanted to know reasons for death	172 (95.6)	0 (0.0)	166 (89.2)	0 (0.0)	115 (57.8)	0 (0.0)	77 (56.6)	0 (0.0)
Mother’s family or in-laws supportive of decision	37 (20.6)	0 (0.0)	7 (3.8)	0 (0.0)	35 (17.6)	0 (0.0)	32 (23.5)	0 (0.0)
No specific reason, but parents were approached by study team	11 (6.1)	0 (0.0)	15 (8.1)	0 (0.0)	66 (33.2)	0 (0.0)	38 (27.9)	0 (0.0)
Other reasons	2 (1.1)	0 (0.0)	3 (1.6)	0 (0.0)	0 (0.0)	0 (0.0)	2 (1.5)	0 (0.0)
Parents and/or relatives given counseling on MITS	180 (100.0)	38 (97.4)	186 (100.0)	66 (89.2)	198 (100.0)	217 (80.1)	136 (100.0)	196 (99.5)
Parents given opportunity to ask questions	179 (99.4)	39 (100.0)	186 (100.0)	74 (100.0)	198 (99.5)	270 (99.6)	136 (100.0)	195 (99.0)
Participants in the decision process								
Mother	158 (87.8)	31 (79.5)	83 (44.6)	41 (55.4)	114 (57.3)	129 (47.6)	23 (16.9)	25 (12.6)
Father	169 (93.9)	37 (94.9)	181 (97.3)	72 (97.3)	138 (69.3)	196 (72.3)	115 (84.6)	133 (67.2)
Father-in-law	3 (1.7)	3 (7.7)	15 (8.1)	8 (10.8)	5 (2.5)	10 (3.7)	8 (5.9)	15 (7.6)
Mother-in-law	12 (6.7)	6 (15.4)	5 (2.7)	9 (12.2)	26 (13.1)	22 (8.1)	15 (11.0)	13 (6.6)
Relatives and others	89 (49.4)	17 (41.0)	45 (24.2)	20 (27.1)	77 (38.7)	116 (42.8)	51 (38.3)	50 (26.3)

Abbreviation: MITS, minimally invasive tissue sampling.

At the Indian site, 39 parents who had a stillbirth and 74 who had a neonatal death refused consent for the MITS procedure. The main reasons for refusing MITS in India, for both stillbirths and neonatal deaths, were cultural concerns (58.1% for stillbirths and 62.7% for preterm neonatal deaths). Among parents consenting to MITS, 172 parents (95.6%) with a stillbirth and 166 (89.2%) with a preterm neonatal death consented to MITS because they wanted to know the cause of the stillbirth or preterm neonatal death. Another reason often given for accepting MITS was that the mother’s family/in-laws were supportive of the decision.

In Pakistan, the decision to allow or refuse the MITS procedure for stillbirths was often made in consultation with the father, mother, and relatives. In India, the decision to allow or refuse the MITS procedure for both stillbirths and preterm neonatal deaths was also made mostly in consultation with the father, mother, and relatives (Table 4).

## Discussion

In the current study, we observed that the MITS procedure for both stillbirths and preterm neonatal deaths was more acceptable to the parents in India than in Pakistan. The high acceptability of MITS in India could be related to the enrollment of study participants from multiple hospitals serving a well-defined catchment area [12]. We assume that community sensitization before the start of the study in the hospital catchment areas and more time given to counseling parents in the hospitals might have provided better opportunities for parents in India to understand the importance of the procedure, thus resulting in better acceptability rates. However, in Pakistan, the study was carried out in a large public sector hospital that serves the entire province, where community sensitization was not possible and the counseling provided to parents was not as rigorously performed [12], perhaps resulting in a lower acceptance rate in Pakistan.

Indian women with a stillbirth or preterm neonatal death who consented to the MITS procedure were younger, more educated, and had received ≥4 ANC visits. In Pakistan, the majority of women who consented to MITS for a stillbirth or a preterm neonatal death were >30 years of age, with the majority having no formal education and not having ANC visits. Women who had a stillbirth and had antepartum hemorrhage in Pakistan were more likely to accept MITS than women with an antepartum hemorrhage in India. Among women who had multiple births, more in Pakistan gave consent for MITS, compared with India.

In a prior qualitative study, both healthcare professionals and parents perceived that parents with a previous history of neonatal deaths, miscarriages, or stillbirths would be more likely to accept the MITS procedure [10, 14]. However, we did not find that these histories affected the likelihood of consent for MITS.

For both India and Pakistan, and for both stillbirths and preterm neonatal deaths, sex, birthweight, and gestational age did not affect parents’ acceptance of MITS. The main persons deciding whether to accept or refuse the MITS procedure were fathers and mothers. In some cases, relatives also played an active role, in both India and Pakistan. The main reason given by parents for consenting to MITS was to know the cause of death in their stillborn or deceased preterm neonate. Similarly, in a study in Kenya, 97% of parents who had agreed to a MITS procedure wanted to know the cause of their child’s death [15].

The main reasons given for refusing MITS in India were mostly cultural concerns, whereas in Pakistan the main reasons for refusal were related to religious prohibition and possible delay in funeral services. However, our prior study found that religious leaders support the MITS procedure if it is done for the benefit of saving other lives, provided that respect is shown for the body and permission is obtained from parents [10].

In India, the main reasons for refusing for MITS after preterm neonatal deaths again related to cultural considerations and parents viewing MITS as unnecessary. In a study from Kenya, parents declined MITS because they thought there was no need for further examination after the death of their child [15]. In Pakistan, however, most women with preterm neonatal deaths who refused MITS noted a delay in the funeral as a reason for refusal. Some women in Pakistan declined MITS owing to lack of trust in the hospital, with a belief that the infant’s organs would be removed. In a study from Bangladesh, the MITS procedure was deemed acceptable because of its shorter duration (compared with CDA) and because there was no removal of organs or cutting and stitching of the body [16].

To the best of our knowledge, this is the first study documenting the frequency and factors influencing MITS acceptance. The data were prospectively collected by trained midwives, with a qualitative study preceding the actual study and with data quality monitored by observing the process of MITS consent.

This study has some limitations. In Pakistan, it was conducted at 2 large public sector tertiary care referral hospitals. Had they a choice, most of the families would have sought care in other healthcare facilities before arriving at the study hospitals and they may have had different attitudes and expectations about understanding the cause of death of their preterm or stillborn child. In India, study participants were provided care in multiple hospitals from well-defined catchment areas. Furthermore, because of the descriptive study design, the cause-and-effect relationship could not be studied.

In conclusion, MITS was more commonly accepted in India than in Pakistan. Cultural concerns in India and funeral delays in Pakistan were common reasons for refusal. Parents from both sites were curious to know the cause of stillbirths and preterm neonatal deaths. Fathers, mothers, and relatives were the key decision makers regarding consent for MITS.

## Notes

***Acknowledgments.*** We acknowledge the senior research assistants for collecting data and the parents who took part in this study. S. S. T. dedicates this article to his late father Professor Pirbhulal Tikmani and late mother Asha Devi (Revti).

***Supplement sponsorship.*** This supplement is sponsored by MITS Surveillance Alliance Secretariat, led by RTI International, with funding from the Bill and Melinda Gates Foundation.

***Financial support.*** This work was supported by the Bill & Melinda Gates Foundation (research grant to RTI International).

***Potential conflicts of interest.*** S. R. reports that the Bill & Melinda Gates Foundation, through Research Triangle Institute, funded the study and will pay the journal for publishing costs. All other authors report no potential conflicts. All authors have submitted the ICMJE Form for Disclosure of Potential Conflicts of Interest. Conflicts that the editors consider relevant to the content of the manuscript have been disclosed.
