# Corrigendum to: Factors Associated With Parental Acceptance of Minimally Invasive Tissue Sampling to Identify the Causes of Stillbirth and Neonatal Death

**DOI:** 10.1093/cid/ciac083

**Published:** 2022-03-17

**Authors:** Shiyam Sunder Tikmani, Sarah Saleem, Janet L Moore, Sayyeda Reza, Guruprasad Gowder, Sangappa Dhaded, S Yogeshkumar, Shivaprasad S Goudar, Vardendra Kulkarni, Sunil Kumar, Anna Aceituno, Lindsay Parlberg, Elizabeth M McClure, Robert L Goldenberg

**Affiliations:** 1 Department of Community Health Sciences, Aga Khan University, Karachi, Pakistan; 2 RTI International, Durham, North Carolina, USA; 3 JJM Medical College, Davengere, India; 4 KLE Academy of Higher Education and Research’s Jawaharlal Nehru Medical College, Belagavi, India; 5 Department of Obstetrics and Gynecology, Columbia University School of Medicine, New York, New York, USA

In the originally published version of this manuscript [Tikmani SS, Saleem S, Moore JL et al. Factors Associated With Parental Acceptance of Minimally Invasive Tissue Sampling to Identify the Causes of Stillbirth and Neonatal Death. Clin. Infect. Dis. Volume 73, Issue Supplement_5, 15 December 2021, Pages S422–S429, https://doi.org/10.1093/cid/ciab829], an author’s name was misspelled as Anna Acetuino. The correct spelling should be Anna Aceituno. The author regrets this error.

